# Landauer’s Principle: Past, Present and Future

**DOI:** 10.3390/e27040437

**Published:** 2025-04-18

**Authors:** Edward Bormashenko

**Affiliations:** Department of Chemical Engineering, Biotechnology and Materials, Engineering Sciences Faculty, Ariel University, Ariel 407000, Israel; edward@ariel.ac.il; Tel.: +972-074-729-68-63

“Thermodynamics is only physical theory of universal content, which I am convinced will never be overthrown, within the framework of applicability of its basic concepts.”

Albert Einstein

The rapid development of computers has led to growing interest in the physical foundations of computation. This interest arises from both applicative and fundamental aspects of computation [1]. It has been hypothesized that the entire universe can be regarded as a giant quantum computer [2]. *Cum grano salis*, even natural evolution can be looked at as a computation that exploits the physical properties of materials [3]. In its most general meaning, computation involves transforming inputs into outputs using a specific set of instructions; we restrict our treatment with a physical framework: a digital computer is seen as physical device, which processes bits by switching logical units “on” and “off”—those physical changes are the computation [4]. A reasonable question that follows from this is what are physical limitations of computation? In other words, what is the minimal energy cost of computation and what is the maximal possible velocity of computation? There are fundamental laws and principles that set the limits of physical systems. Thus, we well expect fundamental limitations on computation to be imposed by nature [5]. From this another question arises: is it possible to break these fundamental limitations under specific circumstances?

Landauer’s principle, addressed in this Special Issue, is one of the limiting physical principles which constrains the behavior of computing systems. There exist fundamental laws and principles that set the limits of physical systems [5,6,7]. These principles include the Abbe diffraction limit [8] and the Heisenberg uncertainty principle [9]. Combining the limiting value of light propagating in a vacuum *c* with the Heisenberg uncertainty principle yields Bremermann’s limit, which enforces a limit on the maximum rate of computation that can be achieved in a self-contained system [10]. Quantum mechanics also gives rise to the Mandelstam–Tamm and Margolus–Levitin limiting principles, which restrict the maximum speed of the dynamical evolution of quantum systems [11,12,13].

Landauer’s principle, in turn, sets a limit the minimum energy necessary for the erasure of one bit of information. Rolf Landauer believed that computation is a physical process; thus, it must obey the laws of physics and, first and foremost, the laws of thermodynamics [14,15,16,17]. This thinking led to a new limiting physical principle by establishing a minimal energy cost for the erasure of a single bit of memory from a system operating at the equilibrium temperature *T*. The minimum amount of heat/energy dissipated when erasing one bit of information is given by

(1) $$W=k_{B}Tln2$$

Landauer’s principle also led to the fundamentally important distinction between logic and thermodynamic irreversibility [18]. It should be emphasized that the Landauer bound, given in Equation (1), relates only to a single information-bearing degree of freedom within an entire computing system. Landauer’s principle was rigorously and microscopically derived without direct reference to the second law of thermodynamics [19]. The quantum mechanics extension of Landauer’s principle has also been demonstrated [20,21]. Elsewhere, the relativistic generalization of Landauer’s principle has been introduced [22,23]. The extension of Landauer’s principle to many-valued logic was addressed in [24].

Combining the Landauer bound with the Margolus–Levitin limiting principle yields the minimal time that it will take for a device to make a single computing operation (as reported in this Special Issue [7]). The minimal “Margolus–Levitin–Landauer” time necessary for a single computation, denoted by $\tau_{MLL}$, is given by Equation (2):

(2) $$\tau_{MLL}\geq \frac{h}{4ln2k_{B}T}=\frac{\tau_{PB}}{4ln2}$$

where $\tau_{PB}=\frac{h}{k_{B}T}$ is the Planck–Boltzmann thermalization time, which is thought to be the fastest relaxation timescale for the thermalization of a given system [25].

Landauer’s principle could be interpreted within the global concept aphoristically called “It from bit”, which was suggested by John Archibald Wheeler. “It from bit symbolizes the idea that every item of the physical world has at bottom … an immaterial source and explanation; that what we call reality arises in the last analysis from the posing of yes-no questions and the registering of equipment-evoked responses; in short, that all things physical are information-theoretic in origin and this is a participatory universe” [26]. Landauer’s principle provides the “It from Bit” idea with measurable physical content by supplying a bridge between “information” and physically measurable values. This bridge was built in a series of recent papers [22,23,27,28,29]. According to Herrera [22], changing one bit of information leads to a decrease in the mass of the system by an amount whose minimal value given by Equation (3):

(3) $$∆M=\frac{k_{B}T}{c^{2}}ln2$$

Generalizations of Landauer’s principle have been reported for logically indeterministic operations and non-equilibrium systems [30,31]. The Landauer bound has been successfully tested in a number of experimental investigations [32,33,34]. Despite this, the meaning and formulation of Landauer’s principle have been intensively criticized. It was argued that since it is not independent of the second law of thermodynamics, it is either a necessary nor sufficient an exorcism of Maxwell’s Demon [35]. Lairez suggested a counterexample with a physical implementation (which uses a two-to-one relation between logic and thermodynamic states) that allows one bit to be erased in a thermodynamic quasi-static manner [36]. Buffoni et al. demonstrated that Landauer’s principle, in contrast to widespread opinion, is not the second law of thermodynamics nor equivalent to it, but in fact a stricter bound [37]. The discussion is far from exhausted. It should be emphasized that real, artificial, and natural computers operate far from thermodynamic equilibrium; thus, the Landauer bound arising from classical equilibrium thermodynamics may be broken [38]. Now, let us briefly list the problems that remain:

(i) The exact place of Landauer’s principle in the structure of thermodynamics should be clarified.
(ii) A relativistic extension of Landauer’s principle remains one of the unsolved problems. The problem of the accurate derivation and grounding of the relativistic transformation of temperature also remains unsolved.
(iii) It is important to implement the Landauer principle in the development of optimal computational protocols, providing minimal energy dissipation, including non-Turing computational devices [39].

## References

[1] Adamatzky A. (2001). Computing in Nonlinear Media and Automata Collectives.

[2] Lloyd S. (2013). The Universe as Quantum Computer, A Computable Universe: Understanding and Exploring Nature as Computation.

[3] Miller J.F., Harding S.L., Tufte G. (2014). Evolution-in-materio: Evolving computation in materials. Evol. Intel..

[4] Piccinini G., Maley C., Zalta E.N. (2010). Computation in physical systems. The Stanford Encyclopedia of Philosophy.

[5] Markov I. (2014). Limits on fundamental limits to computation. Nature.

[6] Liu Y.C., Huang K., Xiao Y.-F., Yang L., Qiu C.W. (2021). What limits limits?. Nat. Sci. Rev..

[7] Bormashenko E. (2024). Landauer Bound in the Context of Minimal Physical Principles: Meaning, Experimental Verification, Controversies and Perspectives. Entropy.

[8] Hecht E. (2002). Modern Optics. Optics.

[9] Landau L.D., Lifshitz E.M. (1977). Quantum Mechanics: Non-Relativistic Theory.

[10] Bremermann H.J., Yovits M.C., Jacobi G.T., Goldstein G.D. (1962). Optimization through evolution and recombination. Self-Organizing Systems 1962.

[11] Mandelstam L., Tamm I., Bolotovskii B.M., Frenkel V.Y., Peierls R. (1991). The Uncertainty Relation Between Energy and Time in Non-Relativistic Quantum Mechanics, in Selected Papers.

[12] Hörnedal N., Sönnerborn O. (2023). Margolus-Levitin quantum speed limit for an arbitrary fidelity. Phys. Rev. Res..

[13] Margolus M., Levitin L.B. (1998). The maximum speed of dynamical evolution. Phys. D Nonlinear Phenomena.

[14] Landauer R. (1961). Dissipation and heat generation in the computing process. IBM J. Res. Dev..

[15] Landauer R. (1991). Information is physical. Phys. Today.

[16] Landauer R. (1996). Minimal energy requirements in communication. Science.

[17] Bennett C.H., Landauer R. (1985). The fundamental physical limits of computation. Sci. Am..

[18] Maroney O.J.E. (2005). The (absence of a) relationship between thermodynamic and logical reversibility. Stud. Hist. Philos. Sci. B.

[19] Piechocinska B. (2000). Information erasure. Phys. Rev. A.

[20] Parrondo J.M.R., Horowitz J.M., Sagawa T. (2015). Thermodynamics of information. Nat. Phys..

[21] Sagawa T. (2014). Thermodynamic and logical reversibilities revisited. J. Stat. Mech..

[22] Herrera L. (2014). The mass of a bit of information and the Brillouin’s principle. Fluct. Noise Lett..

[23] Herrera L. (2020). Landauer Principle and General Relativity. Entropy.

[24] Bormashenko E. (2019). Generalization of the Landauer Principle for Computing Devices Based on Many-Valued Logic. Entropy.

[25] Hartnoll S.A., Mackenzie A.P. (2022). Colloquium: Planckian dissipation in metals. Rev. Mod. Phys..

[26] Wheeler J.A. Information, physics, quantum: The search for links. Proceedings of the 3rd International Symposium on Foundations of Quantum Mechanics in the Light of New Technology.

[27] Vopson M. (2019). The mass-energy-information equivalence principle. AIP Adv..

[28] Müller J.G. (2024). Events as Elements of Physical Observation: Experimental Evidence. Entropy.

[29] Bormashenko E. (2019). The Landauer Principle: Re-Formulation of the Second Thermodynamics Law or a Step to Great Unification?. Entropy.

[30] Maroney O.J.E. (2009). Generalizing Landauer’s principle. Phys. Rev. E.

[31] Esposito M., Van den Broeck C. (2011). Second law and Landauer principle far from equilibrium. Europhys. Lett..

[32] Orlov A.O., Lent C.S., Thorpe C.C., Boechler G.P., Snider G.L. (2012). Experimental Test of Landauer’s Principle at the Sub-*k_B_Y* Level. Jpn. J. Appl. Phys..

[33] Bérut A., Arakelyan A., Petrosyan A., Ciliberto S., Dillenschneider R., Lutz E. (2012). Experimental verification of Landauer’s principle linking information and thermodynamics. Nature.

[34] Jun Y., Gavrilov M., Bechhoefer J. (2014). High-precision test of Landauer’s principle in a feedback trap. Phys. Rev. Lett..

[35] Norton J.D. (2005). Eaters of the lotus: Landauer’s principle and the return of Maxwell’s demon. Stud. Hist. Philos. Sci. B.

[36] Lairez D. (2023). Thermodynamical versus Logical Irreversibility: A Concrete Objection to Landauer’s Principle. Entropy.

[37] Buffoni L., Campisi M. (2022). Spontaneous Fluctuation-Symmetry Breaking and the Landauer Principle. J. Stat. Phys..

[38] Wolpert D.H. (2024). Is stochastic thermodynamics the key to understanding the energy costs of computation?. Proc. Natl. Acad. Sci..

[39] Jaeger H., Noheda B., van der Wiel W.G. (2023). Toward a formal theory for computing machines made out of whatever physics offers. Nat. Commun..

